# Supplementary material for: Species-Level Characterization of the Microbiome in Breast Tissues with Different Malignancy and Hormone-Receptor Statuses Using Nanopore Sequencing
Source: J Pers Med. 2023 Jan 19;13(2):174. doi: 10.3390/jpm13020174 (PMC9965790; doi:10.3390/jpm13020174)
Supplement: Supplementary file 1 [file jpm-13-00174-s001.zip › jpm-2015781-supplementary.pdf]

Supplementary Material:

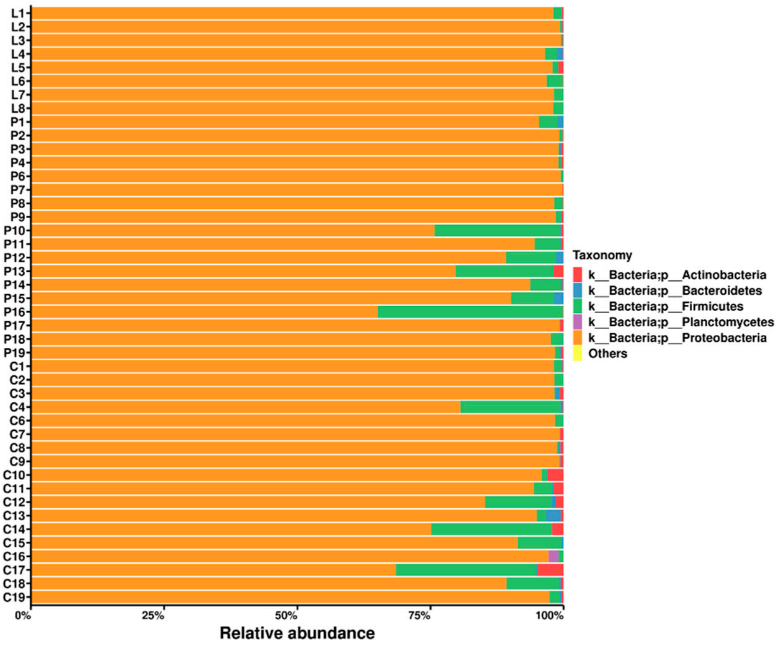

Figure S1. Relative abundance of bacterial phyla within all breast tissues. The left side is marked with the name of the samples, where C1-C19 represent tumor tissues, P1-P19 represent adjacent normal tissues, and L1-L8 represent benign tissues, showing species with relative abundance greater than or equal to 1%.

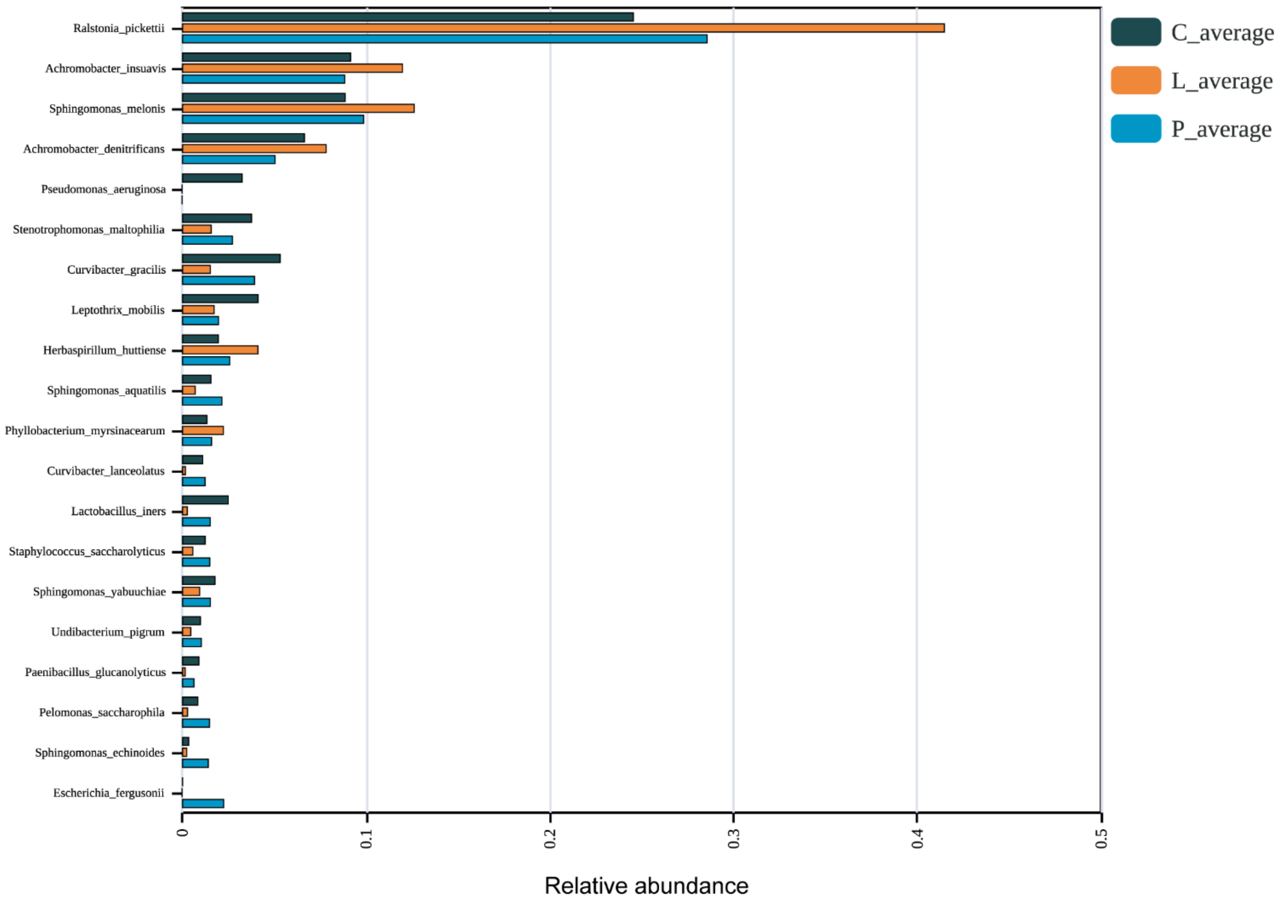

Figure S2. The first 20 species of bacteria with relative abundance fluctuate between benign tissue (L), normal tissue (P), and breast tumor tissue (C), and the length of a colored box represents the relative abundance of that organism within each group.

Table S1: Patient and sample collection characteristics.

Sample	Age	Gender	Type	Grade	Type of tumor	Menopausal status	ER/PR	HER-2
Tumor / Normal Pairs from Same Patient								
C1/P1	62	Female	Malignant	IIA	invasive ductal carcinoma of breast	Post-menopause	(-)	(+)
C2/P2	62	Female	Malignant	IIIC	invasive ductal carcinoma of breast	Post-menopause	(-)	(+)
C3/P3	61	Female	Malignant	IA	invasive ductal carcinoma of breast	Post-menopause	(+)	(-)
C4/P4	62	Female	Malignant	IV	invasive ductal carcinoma of breast	Post-menopause	(-)	(-)
C6/P6	47	Female	Malignant	Missing	invasive ductal carcinoma of breast	Post-menopause	Missing	Missing
C7/P7	42	Female	Malignant	IIB	invasive ductal carcinoma of breast	Pre-menopause	(+)	(+)
C8/P8	49	Female	Malignant	IIIA	invasive ductal carcinoma of breast	Post-menopause	(-)	(-)
C9/P9	46	Female	Malignant	IIA	invasive ductal carcinoma of breast	Post-menopause	(+)	(+)
C10/P10	90	Female	Malignant	IIIB	invasive ductal carcinoma of breast	Post-menopause	(-)	(+)
C11/P11	49	Female	Malignant	III A	invasive ductal carcinoma of breast	Post-menopause	(+)	(+)
C12/P12	55	Female	Malignant	IIB	invasive ductal carcinoma of breast	Post-menopause	(+)	(+)
C13/P13	53	Female	Malignant	IIA	invasive ductal carcinoma of breast	Post-menopause	(-)	(+)
C14/P14	46	Female	Malignant	IIB	invasive ductal carcinoma of breast	Post-menopause	(+)	(+)
C15/P15	57	Female	Malignant	IIA	invasive ductal carcinoma of breast	Post-menopause	(+)	(+)
C16/P16	44	Female	Malignant	IB	invasive ductal carcinoma of breast	Pre-menopause	(+)	(+)
C17/P17	56	Female	Malignant	IIB	invasive ductal carcinoma of breast	Post-menopause	(-)	(+)
C18/P18	43	Female	Malignant	IIIA	invasive ductal carcinoma of breast	Pre-menopause	(+)	(-)
C19/P19	50	Female	Malignant	IB	invasive ductal carcinoma of breast	Post-menopause	(-)	(+)
Benign								
L1	18	Female	Benign	NA	fibroadenoma of breast	Pre-menopause	NA	NA
L2	26	Female	Benign	NA	fibroadenoma of breast	Pre-menopause	NA	NA
L3	29	Female	Benign	NA	benign phyllode tumor of the breast	Pre-menopause	NA	NA
L4	28	Female	Benign	NA	fibroadenoma of breast	Pre-menopause	NA	NA
L5	30	Female	Benign	NA	fibroadenoma of breast	Pre-menopause	NA	NA
L6	35	Female	Benign	NA	intraductal papilloma	Pre-menopause	NA	NA
L7	21	Female	Benign	NA	fibroadenoma of breast	Pre-menopause	NA	NA
L8	19	Female	Benign	NA	atypical hyperplasia	Pre-menopause	NA	NA

Table S2: Raw and processed read counts per sample for sequencing.

Sample ID	Raw Reads	Clean Reads	Clean Base (nt)	AvgLen (nt)	AvgPhred score
L1	40501	40501	59430306	1467	13.8
L2	23512	23512	34530306	1468	13.8
L3	39948	39948	58681850	1469	13.8
L4	30768	30471	44660070	1465	13.8
L5	29510	29310	43012801	1467	13.8
L6	9550	9416	13816237	1467	13.8
L7	18023	17832	26020666	1459	13.8
L8	31703	31547	46330504	1468	13.7
P1	29099	28897	42400470	1467	13.8
P2	18536	18362	26794836	1459	13.7
P3	35036	34922	51187202	1465	13.6
P4	36178	35939	52634198	1464	13.6
P6	20427	20427	29954834	1466	14.2
P7	29656	29656	43375206	1462	14.1
P8	24398	24398	35641326	1460	14.2
P9	21574	21574	31569500	1463	14.1
P10	25898	25898	38184763	1474	14.0
P11	5504	5504	8033195	1459	13.1
P12	136055	136055	199472230	1466	13.5
P13	49379	49379	72360192	1465	13.6
P14	18665	18665	27247179	1459	13.0
P15	11900	11900	17329991	1456	13.1
P16	5426	5426	7993302	1473	13.3
P17	23296	23296	34573144	1484	13.5
P18	10238	10238	14961038	1461	13.1
P19	21889	21889	32021587	1462	12.9
C1	26336	26192	38431187	1467	13.8
C2	27971	27779	40683041	1464	13.8
C3	32673	32444	47530009	1465	13.7
C4	31761	31615	46568145	1473	13.7
C6	24480	24480	35896001	1466	14.2
C7	22577	22577	32951469	1459	14.1
C8	24147	24147	35289706	1461	14.0
C9	30279	30279	44336197	1464	14.0
C10	8412	8412	12307180	1463	14.2
C11	26236	26143	38098067	1457	13.0
C12	78651	78651	115538377	1469	13.5
C13	108997	108997	160721454	1474	13.5
C14	24617	24617	35918019	1459	14.1
C15	12327	12327	18008454	1460	13.0
C16	160943	160943	237163555	1473	13.5
C17	15210	15210	22410374	1473	13.5
C18	13017	13017	19042843	1462	13.0
C19	28694	28694	41986437	1463	13.0

Table S3: Number of different taxa detected in different groups.

Taxon	ALL	Tumor	Benign	Normal pair
Kindom	1	1	1	1
Phylum	8	7	4	6
Class	17	15	11	15
Order	37	32	23	32
Family	86	64	48	69
Genus	250	196	115	177
Species	921	706	387	610
